# Differential Virulence Gene Expression of Group A *Streptococcus* Serotype M3 in Response to Co-Culture with *Moraxella catarrhalis*

**DOI:** 10.1371/journal.pone.0062549

**Published:** 2013-04-23

**Authors:** Suzanne J. C. Verhaegh, Anthony R. Flores, Alex van Belkum, James M. Musser, John P. Hays

**Affiliations:** 1 Department of Medical Microbiology and Infectious Diseases, Erasmus MC, Rotterdam, The Netherlands; 2 Center for Molecular and Translational Human Infectious Diseases Research, The Methodist Hospital Research Institute, Department of Pathology and Genomic Medicine, The Methodist Hospital, Houston, Texas, United States of America; 3 Texas Children’s Hospital and Baylor College of Medicine, Department of Pediatrics, Houston, Texas, United States of America; 4 Microbiology R&D Unit, bioMérieux, La Balme les Grottes, France; Columbia University, United States of America

## Abstract

*Streptococcus pyogenes* (group A *Streptococcus*, GAS) and *Moraxella catarrhalis* are important colonizers and (opportunistic) pathogens of the human respiratory tract. However, current knowledge regarding colonization and pathogenic potential of these two pathogens is based on work involving single bacterial species, even though the interplay between respiratory bacterial species is increasingly important in niche occupation and the development of disease. Therefore, to further define and understand polymicrobial species interactions, we investigated whether gene expression (and hence virulence potential) of GAS would be affected upon co-culture with *M. catarrhalis*. For co-culture experiments, GAS and *M. catarrhalis* were cultured in Todd-Hewitt broth supplemented with 0.2% yeast extract (THY) at 37°C with 5% CO_2_ aeration. Each strain was grown in triplicate so that triplicate experiments could be performed. Bacterial RNA was isolated, cDNA synthesized, and microarray transcriptome expression analysis performed. We observed significantly increased (≥4-fold) expression for genes playing a role in GAS virulence such as hyaluronan synthase (*hasA*), streptococcal mitogenic exotoxin Z (*smeZ*) and IgG endopeptidase (*ideS*). In contrast, significantly decreased (≥4-fold) expression was observed in genes involved in energy metabolism and in 12 conserved GAS two-component regulatory systems. This study provides the first evidence that *M. catarrhalis* increases GAS virulence gene expression during co-culture, and again shows the importance of polymicrobial infections in directing bacterial virulence.

## Introduction

*Streptococcus pyogenes* (group A *Streptococcus*, GAS) is responsible for a wide range of infections including the relatively benign GAS pharyngitis but also life-threatening necrotizing fasciitis and toxic shock syndrome. GAS is also an important cause of post-infectious sequelae such as post-streptococcal glomerulonephritis and rheumatic fever [1], [2], [3], [4]. In contrast, *Moraxella catarrhalis* is a rather exclusive colonizer and primarily opportunistic pathogen of the human respiratory tract, the bacterium being associated with both upper (e.g. otitis media (OM)) and lower (e.g. exacerbations of chronic obstructive pulmonary disease (COPD)) respiratory tract infections. However, both GAS and *M. catarrhalis* share the same respiratory biological niche, particularly with respect to upper respiratory tract infections [5]. For example, although *Streptococcus pneumoniae*, *Haemophilus influenzae* and *M. catarrhalis* are considered the predominant organisms causing OM, studies have shown that GAS may also be included as one of the more frequent causative agents of OM [5], [6]. This includes the development of OM complications such as acute mastoiditis [7], [8]. However, the role of GAS in OM is often underappreciated due to the effectiveness of β-lactam antibiotics in eliminating this organism [9], [10].

Co-colonization by bacterial respiratory pathogens may lead to increased rates of colonization and infection. For example, it has been reported that *M. catarrhalis* and *H. influenzae* enhance the adherence of *S. pyogenes* to human epithelial cells [11], [12]. Further, Armbruster *et al.* (2010) showed that co-colonization of *M. catarrhalis* and *H. influenzae* could influence biofilm formation and antibiotic resistance, while at the same time, Verhaegh *et al.* (2010) showed that co-colonization of infants by *M. catarrhalis* and *H. influenzae* was significantly more likely than single species colonization [13], [14].

Finally, polymicrobial infections may also promote the survival of bacterial species via the phenomenon of ‘indirect pathogenicity’. For example, *S. pneumoniae* is protected from the action of certain β-lactam antibiotics in the presence of BRO β-lactamase positive *M. catarrhalis* [15], [16].Most of the OM knowledge has been derived from work involving single bacterial species, though there is growing evidence for a role for polymicrobial infection. For example, bacteria and viruses are known to cooperate to cause respiratory diseases which are more severe than those caused by either pathogen alone [17].

Given that *M. catarrhalis* and GAS are common bacterial OM pathogens, we hypothesized that polymicrobial infections involving these 2 bacterial pathogens would significantly affect the transcription profile of GAS compared to growth of GAS in isolation.

## Methods

### Bacterial Strains and Growth Conditions

The isolates used in this study were a serotype M3 GAS strain MGAS16655 (cultured from a patient with pharyngitis and closely related to the reference strain MGAS315 [18]), and *M. catarrhalis* strain JMF150 (isolated from a child presenting with OM and provided by Texas Children’s Hospital, Houston, United States). For co-culture experiments, both organisms were cultured in Todd-Hewitt broth supplemented with 0.2% yeast extract (THY) at 37°C with 5% CO_2_ aeration. Further, each strain was grown in triplicate so that triplicate experiments could be performed. In order to quantify viable GAS and *M. catarrhalis*, both in mono-cultures and in co-culture, bacteria were grown in THY broth, and enumerated on the basis of colony forming units (CFU/ml) and OD_600_ readings determined at serial time points.

RNA was extracted from co-cultures of isolates in the exponential/early-stationary phase of growth after an incubation period of 2.5 hours, with control RNA being extracted from MGAS16655 grown under identical but mono-cultural conditions. Two milliliter of co-culture was added to 4 milliliter of RNA protect (Qiagen) and bacteria were incubated at room temperature for 5 minutes, then pelleted by centrifugation at 4000×g at 4°C for 10 minutes, and stored at −80°C until ready for full RNA isolation.

### RNA Isolation

Bacterial RNA was isolated from exponential/early-stationary phase bacterial cells using the QIAGEN RNeasy mini kit according to the manufacturer's instructions. RNA quality was analyzed using an Agilent 2100 bioanalyzer (Agilent Technologies Inc., Palo Alto, CA).

### cDNA Synthesis, Fragmentation, and Labeling

The methods used for cDNA synthesis, fragmentation, and labeling have been described extensively elsewhere [19].

### Microarray Transcriptome Expression Analysis

Microarray expression studies were performed in triplicate using a custom-made Affymetrix GeneChip that contained 100% of the ORFs of the GAS reference strain MGAS315 [20], [21], [22]. Briefly, samples used for microarray analysis were collected from cultures in THY at 2.5 hours after co-culturing. Controls comprised RNA extracted from GAS grown in pure culture under identical conditions. Estimates of gene expression were calculated using GCOS software (Affymetrix) and normalization of data carried out as previously described [23]. Differences in gene expression that were statistically significant (two-sample *t*-test; *P*<0.05) and had a ≥4-fold change in expression were included in the analysis. As a final step, the complete genome of *M. catarrhalis* strain RH4 [24] was compared to the genome of GAS strain MGAS315 for regions of sequence similarity. If a gene was found to be similar in both species using a cut off of >95% similarity, then this gene was removed from the analyses due to potential cross-hybridization of GAS and *M. catarrhalis* RNA transcripts.

### Quantitative Real-Time PCR Analysis

Confirmatory quantitative gene transcript analysis was performed on 2 important GAS virulence associated genes, and the constitutively expressed control gene *proS*, using TaqMan quantitative real-time PCR (qPCR) and an ABI 7500 Fast Real-Time PCR System (Applied Biosystems) as previously described [20], [25], [26]. Transcripts were compared to the internal reference gene *tufA* as previously described [27]. Sequence data for the respective TaqMan primers and probes are listed in Table 1. All reactions were performed in quadruplicate using RNA purified from at least three biological replicates.

**Table 1:** TaqMan quantitative real-time PCR primers and probes utilized in this study.

Gene	Spy no. in strain MGAS315	5′ primer	3′ primer	TaqMan probe
*hasA*	SpyM3_1851	ACCGTTCCCTTGTCAATAAAGG	CGTCAGCGTCAGATCTTTCAAA	CGCCATGCTCAAGCGTGGGC
*proS*	SpyM3_1688	TGAATTTATCATGAAAGACGGCTATAGTTTC	AATAGCTTCGTAAGCTTGACGATAATC	TCGTAGGTCACATCTAAATCTTCATAGTTG
*speB*	SpyM3_1742	CGCACTAAACCCTTCAGCTCTT	ACAGCACTTTGGTAACCGTTGA	GCCTGCGCCGCCACCAGTA

## Results

### Characterization of Serotype M3 GAS Strain MGAS16655 and *M. catarrhalis* JMF150 Growth as Mono-cultures and in Co-culture

**Figure 1:**
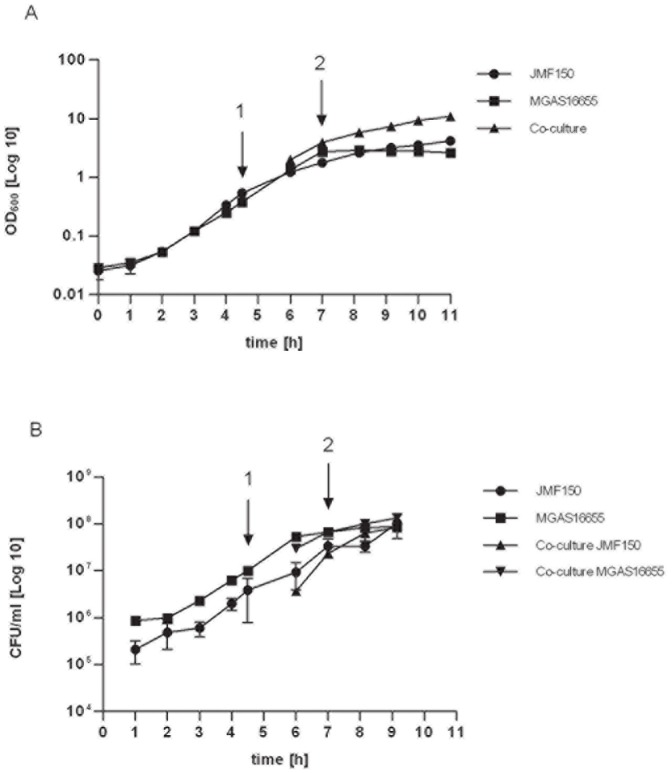
Characterization of growth curves for GAS serotype M3 MGAS16655 [▪], *M. catarrhalis* isolate JMF150 [•], and co-culture [▴ or ▾]. Bacterial growth was measured over time in 3 independent experiments by determination of A) the OD_600_ and B) the CFU/ml, at hourly time points. The growth curve displayed represents the mean OD_600_ and CFU/ml values. Error bars indicate the standard deviation of the mean between individual experiments. Sampling time points for the start of co-culture [1] and for the harvesting of cells for RNA-isolation [2] are indicated by arrows.

Prior to characterizing the transcriptome of GAS, we studied the growth of strains MGAS16655 and JMF150 in THY medium in co-culture and as pure cultures. Both strains grew rapidly in this medium, and the bacterial density reached ∼10^8^ CFU/ml in the exponential/early-stationary phase (Figure 1). Further, these experiments showed that we were able to reproducibly correlate OD_600_ readings to CFU concentrations for both MGAS16655 and JMF150.

### Changes in the GAS Transcriptome Induced by Co-culture with *M. catarrhalis*

**Figure 2:**
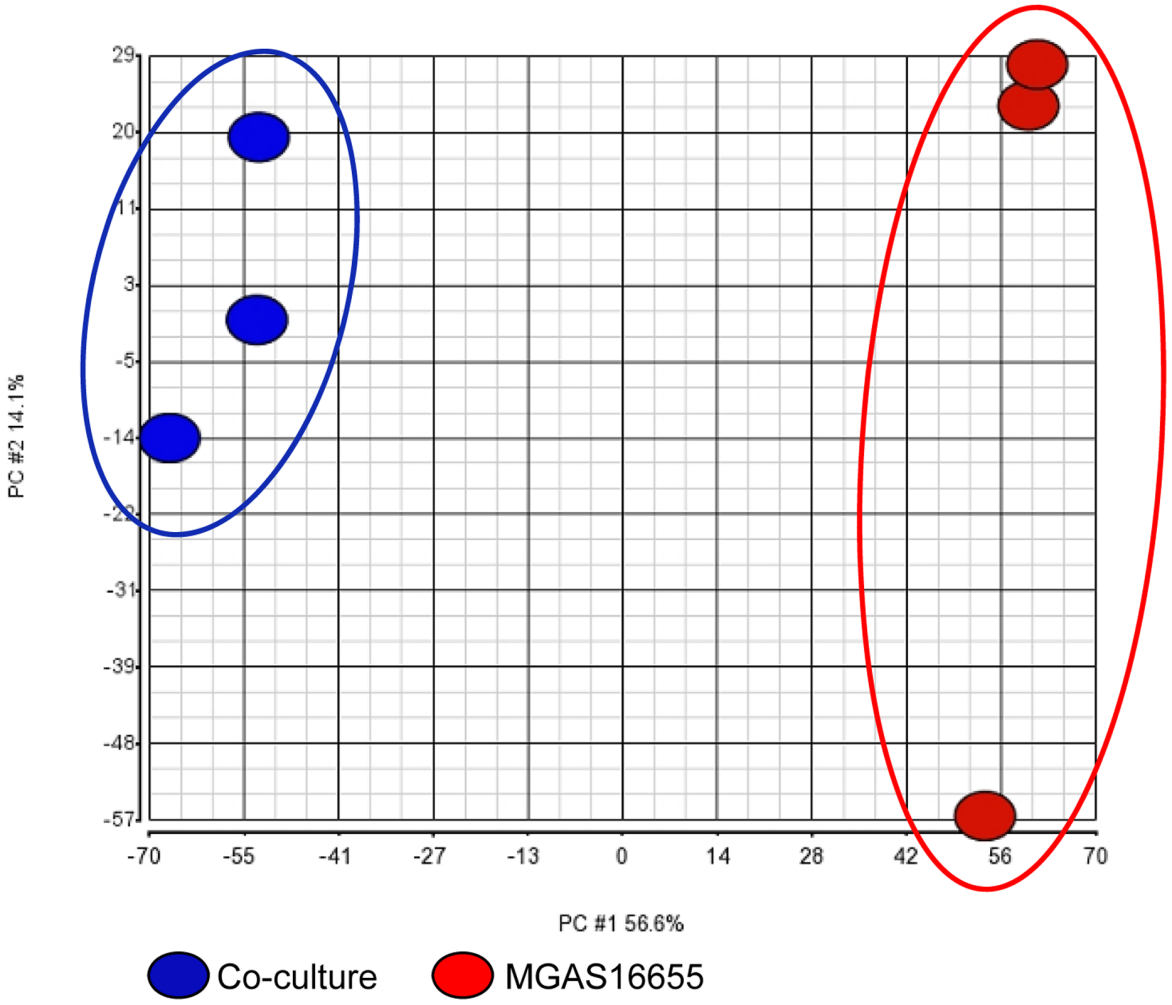
Principal component analysis (PCA) indicating the two different growth conditions (co-culture versus mono-culture) generate distinct GAS gene transcription profiles.

Microarray expression analysis was used to test the hypothesis that GAS gene transcript levels differed significantly during growth in co-culture with *M. catarrhalis* compared to GAS pure culture alone. Principal component analysis (PCA) was performed (Figure 2) and indicated that the two different growth conditions (co-culture versus mono-culture) generated distinct GAS gene transcription profiles, with multiple virulence and energy metabolism genes being differentially expressed upon co-culture compared to the constitutively expressed control gene *proS* (Table 2).

**Table 2:** Top 15 up- and downregulated GAS serotype M3 MGAS16655 genes with ≥4-fold difference in expression during co-culture with *M. catarrhalis* compared to GAS serotype M3 culture alone.

Spy no. in strain MGAS315	Fold change	Gene	Function
SpyM3_1851	181.321	*hasA*	Hyaluronan synthase
SpyM3_0616	34.675	*pyrF*	Orotidine phosphate decarboxylase
SpyM3_0617	29.003	*pyrE*	Orotate phosphoribosyltransferase
SpyM3_0558	26.203	*pyrR*	Uracil phosphoribosyltransferase/Pyrimidine operon regulatory protein PyrR
SpyM3_0559	24.299	*pyrP*	Uracil permease
SpyM3_1716	22.044	–	Streptococcal mitogenic exotoxin Z (SmeZ)
SpyM3_0132	20.776	–	hypothetical protein
SpyM3_1853	20.445	*hasC*	UTP-glucose-1-phosphate uridylyltransferase
SpyM3_0777	19.294	*radC*	DNA repair protein RadC
SpyM3_1543	16.992	–	Nicotinamidase
SpyM3_0618	15.182	*amiC*	6-aminohexanoate-cyclic-dimer hydrolase
SpyM3_0561	13.653	*carA*	Carbamoyl-phosphate synthase small chain
SpyM3_0583	12.703	–	Immunoglobulin G endopeptidase (IdeS)/Secreted immunoglobulin binding protein (Sib38)
SpyM3_0560	12.479	*pyrB*	Aspartate carbamoyltransferase
SpyM3_0562	11.733	–	–
SpyM3_1652	−49.579	*salA*	Lantibiotic salivaricin A
SpyM3_1655	−51.635	*lacF*	PTS system, lactose-specific IIA component
SpyM3_1678	−53.819	*ulaA*	Transport protein SgaT, putative
SpyM3_1657	−54.741	*lacC.2*	Tagatose-6-phosphate kinase (EC 2.7.1.144)/1-phosphofructokinase
SpyM3_1482	−58.292	*lacD.1*	Tagatose 1,6-diphosphate aldolase
SpyM3_1486	−78.036	–	PTS system, galactose-specific IIC component
SpyM3_1742	−79.421	*speB*	Strepotococcal cysteine protease (Streptopain)/Streptococcal pyrogenic exotoxin B (SpeB)
SpyM3_1484	−84.780	*lacB.1*	Galactose-6-phosphate isomerase, LacB subunit
SpyM3_1679	−93.265	–	PTS system IIB component
SpyM3_1656	−106.265	*lacD.2*	Tagatose 1,6-diphosphate aldolase
SpyM3_1659	−109.750	*lacA.2*	Galactose-6-phosphate isomerase, LacA subunit
SpyM3_1485	−125.047	*lacA.1*	Galactose-6-phosphate isomerase, LacA subunit
SpyM3_1751	−162.809	–	PTS system, cellobiose-specific IIB component
SpyM3_1680	−371.559	–	Transcription antiterminator, BglG family
SpyM3_1677	−431.501	–	Transaldolase

**Figure 3:**
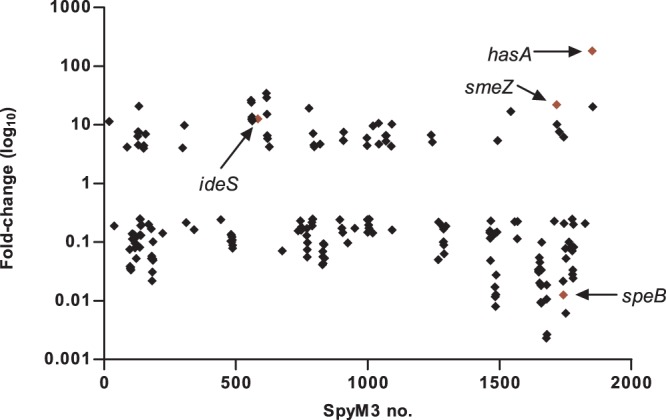
Genes showing significant changes in expression, using a 4-fold cut-off value, for GAS serotype M3 MGAS16655 when grown in co-culture with *M. catarrhalis*. Results were calculated relative to gene expression values obtained for GAS M3 MGAS16655 grown in pure culture alone. One hundred and ninety-two genes were either 4-fold increased (52 genes) or 4-fold decreased (140 genes) in expression, which corresponds to 10% of the MGAS315 genome. The *hasA*, *ideS*, *smeZ* and *speB* genes have been associated with GAS virulence.

GAS transcription expression levels were affected by a factor of at least 4-fold or greater for a total of 207 genes (61 genes with increased expression and 147 genes with decreased expression), after growth in co-culture and relative to the growth of GAS alone. Further, BLAST searching of whole genome sequences revealed that only a small percentage of the *M. catarrhalis* RNA transcripts were predicted to cross-hybridize with the GAS-specific microarray. When these potentially cross-hybridizing genes (*n* = 15) were subtracted from the list of statistically significant, ≥4-fold, GAS expression transcripts, 192 genes remained. The final list comprised 52 genes with significantly increased expression and 140 genes with significantly decreased expression (Figure 3).

**Figure 4:**
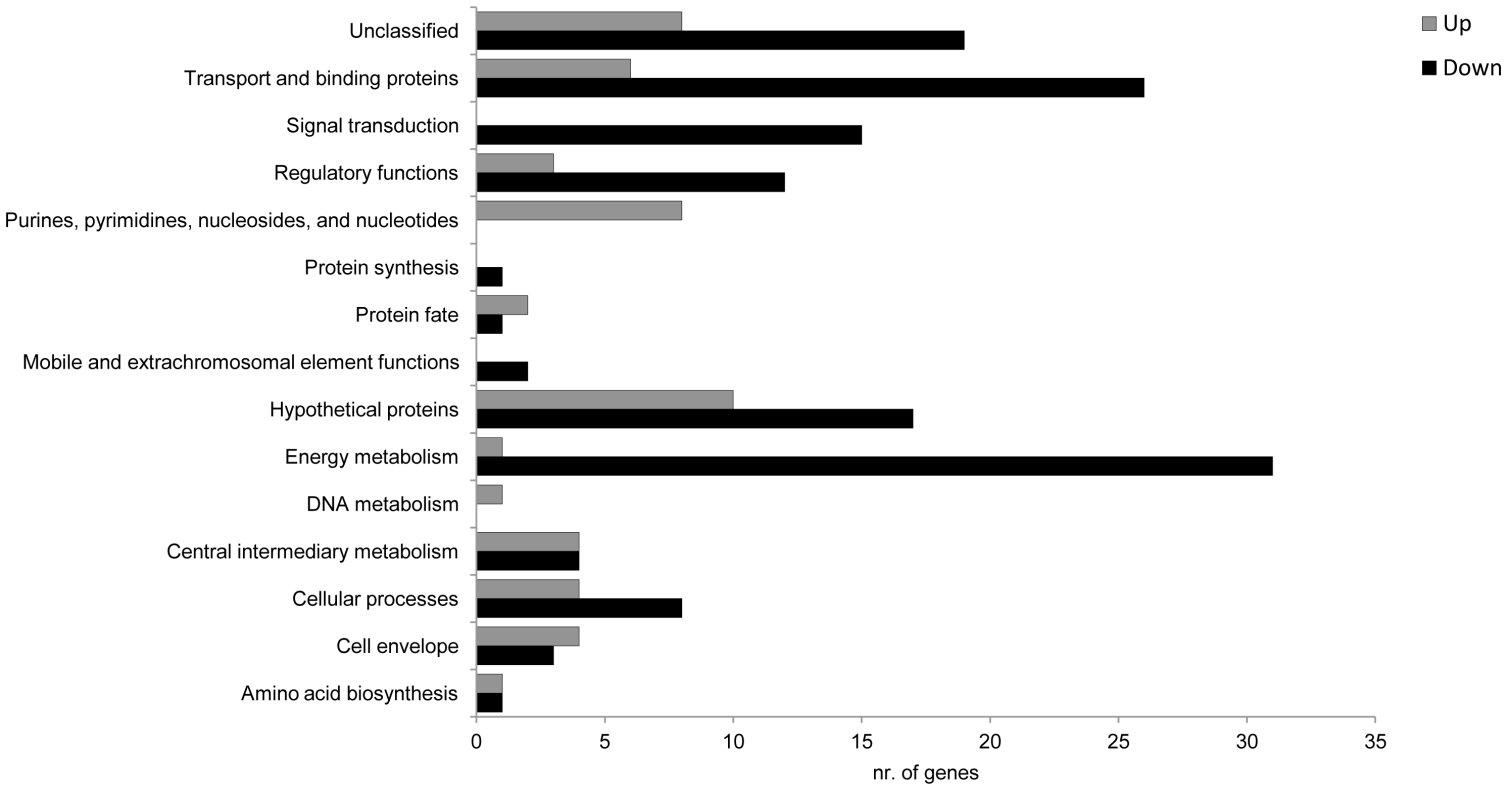
Number of genes up- or down-regulated after GAS co-culture with *M. catarrhalis*. Genes were classified into 14 main functional categories. Genes associated with energy metabolism comprised the most frequent down-regulated genes.

Significant down-regulation was observed for many genes of the functional subcategories as shown in Figure 4 (73% of the genes whose expression was modified were down-regulated in co-culture relative to mono-culture), for example genes with products involved in signal transduction tended to be down-regulated. In contrast, genes encoding proteins involved in purine/pyrimidine metabolism were generally up-regulated. A key finding was that a ∼180-fold increase and ∼80-fold decrease in the expression of hyaluronic synthase (*hasA*) and streptococcal pyrogenic exotoxin B (*speB*) was observed, along with a significant decrease in the expression of many genes involved in carbohydrate utilization. Further, gene expression in co-culture resulted in significant differences in several GAS two-component systems (TCS) and response regulatory systems (Table 3), compared to the results observed for GAS pure culture alone.

**Table 3:** Fold-change in TCS transcripts in MGAS16655 grown in co-culture with *M. catarrhalis* and relative to MGAS16655 grown as a pure culture.

Spy no. in strain MGAS315	Fold-change	Two-component systems in GAS genomes
SpyM3_0174	−2.807	*fasB-fasC-fasA*
SpyM3_0244–0245	<2	*covR-covS*
SpyM3_0372–0373	<2	
SpyM3_0594	−2.201	
SpyM3_0745–0746	−3.702/−4.342	
SpyM3_0768–0769	−5.821/−7.705	
SpyM3_0873	−2.336	
SpyM3_1201–1202	−2.501/−2.763	
SpyM3_1286–1287	−5.951/−9.907	*trxRS*
SpyM3_1366–1367	<2	*yvqCE*
SpyM3_1645–1646	−2.686/−2.710	
SpyM3_1732–1733	−2.270/−3.049	*ihk-irr*

### Validation of DNA Microarray Data

TaqMan quantitative real-time reverse transcription PCR analysis confirmed the validity of the DNA microarray results for 2 representative and significantly up- or down-regulated virulence genes, and the constitutively expressed control gene *proS* (Table 4).

**Table 4:** TaqMan validation for co-culture versus pure culture for hyaluronan synthase (*hasA*), prolyl-tRNA synthase (*proS*), and cysteine protease (*speB*).

Gene	Spy no. in strain MGAS315	Fold-change co-culture (MGAS16655 Array)	Fold-change co-culture (MGAS16655 TaqMan)
*hasA*	SpyM3_1851	181.321	472.499
*proS*	SpyM3_1688	−1.916	−2.244
*speB*	SpyM3_1742	−79.421	−75116.037

## Discussion

The primary goal of this study was to investigate the changes in GAS gene expression in co-culture with *M. catarrhalis*. We discovered 192 GAS genes to be significantly affected (≥4-fold difference). Several of the most significantly up-regulated genes during co-culture were known virulence genes (hyaluronan synthase, streptococcal mitogenic exotoxin Z and IgG endopeptidase), as well as genes involved in purine and pyrimidine metabolism. However, we also observed significant down-regulation in genes involved in energy metabolism and regulatory two-component systems. The hyaluronic acid capsule (hyaluronan synthase gene *hasA*) contributes to resistance to phagocytosis, bacterial aggregation, biofilm maturation, resistance to host defense peptides and neutrophil extracellular killing [28]. GAS also produces a number of highly potent exotoxins that act as superantigens. The streptococcal mitogenic exotoxin Z (SmeZ) is the most potent bacterial superantigen so far discovered and enhances the cascade of pro-inflammatory events that follow invasive streptococcal infection [29], [30]. GAS also secretes a highly effective IgG endopeptidase (IdeS) that inhibits phagocytic killing by cleavage of specific IgG [31], [32], [33]. Finally, though not listed in the top 15 up- and downregulated GAS serotype M3 MGAS16655 genes, the virulence factors streptolysin O (SLO) and streptolysin S (SLS) showed significant differences in expression during co-culture. However, the expression of SLO and SLS are both growth phase dependent; SLO being expressed during the exponential and early stationary-phase of GAS growth, with SLS expression occurring when cells are in the stationary-phase [34], [35]. The fact that mRNA was extracted during the exponential/early-stationary phase of co-culture in this manuscript explains why in our study, SLO was upregulated 4.5-fold and the nine-gene locus (sagA-sagI) that is associated with SLS production was downregulated >3-fold. That the transcripts of 12 of the 13 TCS in our study were significantly affected suggests a common signal or significant cross-talk between GAS TCS. TCS are integral in the regulation of the expression of many key virulence factors and serve as a basic stimulus-response coupling mechanism to allow organisms to sense and respond to changes in many different environmental conditions. Examination of whole genome sequence data revealed 13 TCS [36]; however, only 3 GAS TCS have been studied in detail with respect to their involvement in host-pathogen interactions [23], [36]. Our data showed down-regulation of the 12 conserved GAS TCS, a finding which when taken in context with the concomitant upregulation of virulence genes, shows that: i) TCS respond to changes in the GAS environment, ii) TCS may be involved in the regulation of GAS pathogenesis, iii) TCS may exert a growth phase-associated control over GAS virulence factors, and iv) GAS TCS may be susceptible to *M. catarrhalis* regulatory signals [21], [37].

Our transcriptomics data are particularly relevant in the context of recent research detailing the effect of quorum sensing on the polymicrobial interactions of other respiratory bacterial pathogens. In particular, a recent study by Armbruster *et al.* (2010) showed that co-colonization of *M. catarrhalis* and *H. influenzae* (bacterial pathogens sharing the same respiratory niche as GAS) results in increased *M. catarrhalis* biofilm formation and resistance to antibiotics. This process was achieved via an autoinducer-2 (AI-2, *luxS*) quorum sensing system [13]. At the same time, Verhaegh *et al.* (2010), showed that co-colonization of *M. catarrhalis* and *H. influenzae* was significantly more likely than single species colonization with either *M. catarrhalis* or *H. influenzae* alone, adding clinical epidemiological evidence to the laboratory-based findings [14]. In fact, many species of bacteria use quorum sensing systems to coordinate their gene expression and biofilm formation, dependent on the local bacterial cell density. A homolog of *luxS*, the genetic determinant for AI-2 production, has been identified in GAS, though a *luxS* homolog in *M. catarrhalis* has not been found. However, evidence from whole genome sequencing does suggest that *M. catarrhalis* possesses an AI-1 *luxR* sensing system, but no quorum sensing system [38]. Chang *et al.* (2011) reported that the Rgg family of transcriptional regulators function as quorum-sensing effector proteins and comprise the first functional quorum-sensing pathway conserved in all group A streptococci [39]. Expression of SpeB is dependent upon this regulator, and is upregulated with increasing cell density and during infection, although Rgg does not appear to be mediator of growth phase regulated control [40]. Currently however, a role for quorum sensing systems in facilitating the observed GAS - *M. catarrhalis* polymicrobial interaction is still open to question, not least due to the fact that less ‘targeted’ mechanisms could be responsible for the change in GAS transcriptome profile during co-culture, including competition for nutrients.

The 15 genes that showed the most significant decrease in expression during co-culture were mainly involved in energy metabolism, with an emphasis on galactose metabolism. Nutrient availability is a signal by which pathogens sense their external environment and to which they respond through regulated production of various virulence factors. Studies have shown that co-cultured bacteria compete for nutrients and that interactions between co-cultured strains also involve species-specific pH limits for growth and differential utilization of growth substrates [41], [42]. The success of GAS, which thrives in diverse host niches, depends on the acquisition of nutrients from very different sources and GAS virulence gene expression is highly responsive to carbohydrate source and availability; however, the pathways linking metabolism and virulence remain poorly understood [20], [43]. Our findings could also be associated with a GAS transcriptional response to depletion of carbohydrate (and possible other nutrients) within the polymicrobial environment, thereby helping GAS to conserve its ‘energy reserves’, and ultimately facilitate entry into a sessile ‘state of rest’ (associated with biofilm formation) [44]. However, it should be noted that *M. catarrhalis* is biochemically a-saccharolytic, meaning that it is unable to metabolize glucose or other carbohydrates [45]. Therefore, the true relationship between *M. catarrhalis* and GAS co-culture, carbohydrate depletion, and the down-regulation of genes involved in carbohydrate utilization remains to be further elucidated.

One gene that showed a significant decrease in expression during co-culture, and is not involved in energy metabolism, was *salA* (salivaricin A). Salivaricin A is an antimicrobial peptide which is a member of the class of antimicrobial peptides called lantibiotics. Lantibiotics are produced by a large number of Gram-positive bacteria such as *Streptococcus* and *Streptomyces* to attack other Gram-positive bacteria. A study by Upton *et al.* (2001) described intra- and interspecies signaling between *Streptococcus salivarius* and *S. pyogenes*, mediated by SalA [46]. A single bacteriocin (McbC) has been described in *M. catarrhalis*, though the effect of this bacteriocin on GAS growth and virulence is not yet known [47].

Currently, watchful waiting and/or the prescription of antibiotics are the most favored options for the treatment of organisms causing OM disease. Treatment with antibiotics seems effective, but is not favorable due to the development of antibiotic resistance. Targeting bacterial communication (quorum signaling) could be a novel treatment methodology, as there is growing evidence for a role for polymicrobial infection in de development of OM. For example, *M. catarrhalis* and GAS together could generate a more severe infection than *M. catarrhalis* or GAS alone. Further, *M. catarrhalis* outer membrane vesicles (OMVs) provide protection to *S. pneumoniae* and *H. influenzae* by carrying active β-lactamase [48]. Many bacteria rely on quorum signaling molecules to control the expression of virulence and regulatory genes, which ultimately influence the establishment and progress of disease. However, the interspecies targeting of quorum signaling molecules may result in undesirable complications, such as the promotion of virulence or virulence-related phenotypes in opportunistic pathogens that share the same biological niche as *S. pyogenes* [49].

### Conclusion

The transcriptome profile of GAS is markedly altered in response to co-culture with *M. catarrhalis*, with genes involved in virulence (up-regulated) and carbohydrate utilization (down-regulated) being especially affected. Though the study findings are limited due the use of one strain per species and the lack of protein verification assays, our findings are a first step in understanding and elucidating the mechanisms that help facilitate colonization and disease during polymicrobial infections with GAS and *M. catarrhalis*.

## References

[1] Cunningham MW (2000) Pathogenesis of group A streptococcal infections. Clin Microbiol Rev 13: 470–511. 10885988 10.1128/cmr.13.3.470-511.2000 PMC88944

[2] Cunningham MW (2008) Pathogenesis of group A streptococcal infections and their sequelae. Adv Exp Med Biol 609: 29–42. 18193655 10.1007/978-0-387-73960-1_3

[3] Garcia-Casares E, Mateo Soria L, Garcia-Melchor E, Riera Alonso E, Olive Marques A, et al (2010) Necrotizing fasciitis and myositis caused by streptococcal flesh-eating bacteria. J Clin Rheumatol 16: 382–384. 21085016 10.1097/RHU.0b013e3181fe8ba3

[4] Johansson L, Thulin P, Low DE, Norrby-Teglund A (2010) Getting under the skin: the immunopathogenesis of *Streptococcus pyogenes* deep tissue infections. Clin Infect Dis 51: 58–65. 20491545 10.1086/653116

[5] Vergison A (2008) Microbiology of otitis media: a moving target. Vaccine 26 Suppl 7G5–10. 19094935 10.1016/j.vaccine.2008.11.006 PMC7127463

[6] Torretta S, Marchisio P, Drago L, Baggi E, De Vecchi E, et al (2012) Nasopharyngeal biofilm-producing otopathogens in children with nonsevere recurrent acute otitis media. Otolaryngol Head Neck Surg 146: 991–996. 22357644 10.1177/0194599812438169

[7] Marchisio P, Bianchini S, Capaccio P, Esposito S, Fusi M, et al (2010) Insights into infectious otitis media. Int J Immunopathol Pharmacol 23: 20–23. 20152074

[8] Stahelin-Massik J, Podvinec M, Jakscha J, Rust ON, Greisser J, et al (2008) Mastoiditis in children: a prospective, observational study comparing clinical presentation, microbiology, computed tomography, surgical findings and histology. Eur J Pediatr 167: 541–548. 17668240 10.1007/s00431-007-0549-1

[9] Roberts AL, Connolly KL, Doern CD, Holder RC, Reid SD (2010) Loss of the group A *Streptococcus* regulator Srv decreases biofilm formation in vivo in an otitis media model of infection. Infect Immun 78: 4800–4808. 20805338 10.1128/IAI.00255-10 PMC2976359

[10] Traub WH, Leonhard B (1997) Comparative susceptibility of clinical group A, B, C, F, and G beta-hemolytic streptococcal isolates to 24 antimicrobial drugs. Chemotherapy 43: 10–20. 8996736 10.1159/000239529

[11] Lafontaine ER, Wall D, Vanlerberg SL, Donabedian H, Sledjeski DD (2004) *Moraxella catarrhalis* coaggregates with *Streptococcus pyogenes* and modulates interactions of *S. pyogenes* with human epithelial cells. Infect Immun 72: 6689–6693. 15501804 10.1128/IAI.72.11.6689-6693.2004 PMC523028

[12] Xu Q, Pichichero ME, Zeng M (2009) Adherence of *Streptococcus pyogenes* to human epithelial cells is modulated by *Haemophilus influenzae* . Scand J Infect Dis 41: 244–251. 19214868 10.1080/00365540902767064

[13] Armbruster CE, Hong W, Pang B, Weimer KE, Juneau RA, et al. (2010) Indirect pathogenicity of *Haemophilus influenzae* and *Moraxella catarrhalis* in polymicrobial otitis media occurs via interspecies quorum signaling. MBio 1: pii: e00102–00110. 10.1128/mBio.00102-10 PMC2925075 20802829

[14] Verhaegh SJ, Snippe ML, Levy F, Verbrugh HA, Jaddoe VW, et al (2011) Colonization of healthy children by *Moraxella catarrhalis* is characterized by genotype heterogeneity, virulence gene diversity and co-colonization with *Haemophilus influenzae* . Microbiology 157: 169–178. 20847012 10.1099/mic.0.042929-0

[15] Budhani RK, Struthers JK (1998) Interaction of *Streptococcus* pneumoniae and *Moraxella catarrhalis*: investigation of the indirect pathogenic role of beta-lactamase- producing moraxellae by use of a continuous-culture biofilm system. Antimicrob Agents Chemother 42: 2521–2526. 9756750 10.1128/aac.42.10.2521 PMC105877

[16] Hol C, Van Dijke EE, Verduin CM, Verhoef J, van Dijk H (1994) Experimental evidence for *Moraxella*-induced penicillin neutralization in pneumococcal pneumonia. J Infect Dis 170: 1613–1616. 7996007 10.1093/infdis/170.6.1613

[17] Massa HM, Cripps AW, Lehmann D (2009) Otitis media: viruses, bacteria, biofilms and vaccines. Med J Aust 191: S44–49. 19883356 10.5694/j.1326-5377.2009.tb02926.x PMC7168357

[18] Shea PR, Beres SB, Flores AR, Ewbank AL, Gonzalez-Lugo JH, et al (2011) Distinct signatures of diversifying selection revealed by genome analysis of respiratory tract and invasive bacterial populations. Proc Natl Acad Sci U S A 108: 5039–5044. 21383167 10.1073/pnas.1016282108 PMC3064369

[19] Mereghetti L, Sitkiewicz I, Green NM, Musser JM (2008) Extensive adaptive changes occur in the transcriptome of *Streptococcus agalactiae* (group B *Streptococcus*) in response to incubation with human blood. PLoS One 3: e3143. 18769548 10.1371/journal.pone.0003143 PMC2519835

[20] Shelburne SA 3rd, Keith D, Horstmann N, Sumby P, Davenport MT, et al (2008) A direct link between carbohydrate utilization and virulence in the major human pathogen group A *Streptococcus* . Proc Natl Acad Sci U S A 105: 1698–1703. 18230719 10.1073/pnas.0711767105 PMC2234207

[21] Sitkiewicz I, Green NM, Guo N, Bongiovanni AM, Witkin SS, et al (2009) Transcriptome adaptation of group B *Streptococcus* to growth in human amniotic fluid. PLoS One 4: e6114. 19568429 10.1371/journal.pone.0006114 PMC2700258

[22] Sitkiewicz I, Green NM, Guo N, Bongiovanni AM, Witkin SS, et al (2010) Adaptation of group A *Streptococcus* to human amniotic fluid. PLoS One 5: e9785. 20352104 10.1371/journal.pone.0009785 PMC2843714

[23] Sumby P, Whitney AR, Graviss EA, DeLeo FR, Musser JM (2006) Genome-wide analysis of group a streptococci reveals a mutation that modulates global phenotype and disease specificity. PLoS Pathog 2: e5. 16446783 10.1371/journal.ppat.0020005 PMC1354197

[24] de Vries SP, van Hijum SA, Schueler W, Riesbeck K, Hays JP, et al (2010) Genome analysis of *Moraxella catarrhalis* strain RH4, a human respiratory tract pathogen. J Bacteriol 192: 3574–3583. 20453089 10.1128/JB.00121-10 PMC2897349

[25] Virtaneva K, Graham MR, Porcella SF, Hoe NP, Su H, et al (2003) Group A *Streptococcus* gene expression in humans and cynomolgus macaques with acute pharyngitis. Infect Immun 71: 2199–2207. 12654842 10.1128/IAI.71.4.2199-2207.2003 PMC152081

[26] Shelburne SA 3rd, Okorafor N, Sitkiewicz I, Sumby P, Keith D, et al (2007) Regulation of polysaccharide utilization contributes to the persistence of group A *Streptococcus* in the oropharynx. Infect Immun 75: 2981–2990. 17403878 10.1128/IAI.00081-07 PMC1932865

[27] Virtaneva K, Porcella SF, Graham MR, Ireland RM, Johnson CA, et al (2005) Longitudinal analysis of the group A *Streptococcus* transcriptome in experimental pharyngitis in cynomolgus macaques. Proc Natl Acad Sci U S A. 102: 9014–9019. 10.1073/pnas.0503671102 PMC1150296 15956184

[28] Cole JN, Pence MA, von Kockritz-Blickwede M, Hollands A, Gallo RL, et al. (2010) M protein and hyaluronic acid capsule are essential for in vivo selection of covRS mutations characteristic of invasive serotype M1T1 group A *Streptococcus*. MBio 1. 10.1128/mBio.00191-10 PMC2934611 20827373

[29] Sriskandan S, Faulkner L, Hopkins P (2007) *Streptococcus pyogenes*: Insight into the function of the streptococcal superantigens. Int J Biochem Cell Biol 39: 12–19. 17029999 10.1016/j.biocel.2006.08.009

[30] Unnikrishnan M, Altmann DM, Proft T, Wahid F, Cohen J, et al (2002) The bacterial superantigen streptococcal mitogenic exotoxin Z is the major immunoactive agent of *Streptococcus pyogenes* . J Immunol 169: 2561–2569. 12193726 10.4049/jimmunol.169.5.2561

[31] Berggren K, Johansson B, Fex T, Kihlberg J, Bjorck L, et al (2009) Synthesis and biological evaluation of reversible inhibitors of IdeS, a bacterial cysteine protease and virulence determinant. Bioorg Med Chem 17: 3463–3470. 19362485 10.1016/j.bmc.2009.03.026

[32] Soderberg JJ, von Pawel-Rammingen U (2008) The streptococcal protease IdeS modulates bacterial IgGFc binding and generates 1/2Fc fragments with the ability to prime polymorphonuclear leucocytes. Mol Immunol 45: 3347–3353. 18533265 10.1016/j.molimm.2008.04.013

[33] von Pawel-Rammingen U, Bjorck L (2003) IdeS and SpeB: immunoglobulin-degrading cysteine proteinases of *Streptococcus pyogenes* . Curr Opin Microbiol 6: 50–55. 12615219 10.1016/s1369-5274(03)00003-1

[34] Sierig G, Cywes C, Wessels MR, Ashbaugh CD (2003) Cytotoxic effects of streptolysin o and streptolysin s enhance the virulence of poorly encapsulated group A streptococci. Infect Immun 71: 446–455. 12496195 10.1128/IAI.71.1.446-455.2003 PMC143243

[35] Alouf JE (1980) Streptococcal toxins (streptolysin O, streptolysin S, erythrogenic toxin). Pharmacol Ther 11: 661–717. 7003609 10.1016/0163-7258(80)90045-5

[36] Sitkiewicz I, Musser JM (2006) Expression microarray and mouse virulence analysis of four conserved two-component gene regulatory systems in group a *Streptococcus* . Infect Immun 74: 1339–1351. 16428783 10.1128/IAI.74.2.1339-1351.2006 PMC1360370

[37] Kreikemeyer B, Boyle MD, Buttaro BA, Heinemann M, Podbielski A (2001) Group A streptococcal growth phase-associated virulence factor regulation by a novel operon (Fas) with homologies to two-component-type regulators requires a small RNA molecule. Mol Microbiol 39: 392–406. 11136460 10.1046/j.1365-2958.2001.02226.x

[38] Wang W, Reitzer L, Rasko DA, Pearson MM, Blick RJ, et al (2007) Metabolic analysis of *Moraxella catarrhalis* and the effect of selected in vitro growth conditions on global gene expression. Infect Immun 75: 4959–4971. 17620351 10.1128/IAI.00073-07 PMC2044516

[39] Chang JC, LaSarre B, Jimenez JC, Aggarwal C, Federle MJ (2011) Two group A streptococcal peptide pheromones act through opposing Rgg regulators to control biofilm development. PLoS Pathog 7: e1002190. 21829369 10.1371/journal.ppat.1002190 PMC3150281

[40] Shelburne Iii SA, Olsen RJ, Makthal N, Brown NG, Sahasrabhojane P, et al (2011) An amino-terminal signal peptide of Vfr protein negatively influences RopB-dependent SpeB expression and attenuates virulence in *Streptococcus pyogenes* . Mol Microbiol 82: 1481–95. 22040048 10.1111/j.1365-2958.2011.07902.x PMC4571278

[41] Leriche V, Carpentier B (2000) Limitation of adhesion and growth of *Listeria monocytogenes* on stainless steel surfaces by *Staphylococcus sciuri* biofilms. J Appl Microbiol 88: 594–605. 10792517 10.1046/j.1365-2672.2000.01000.x

[42] Mellefont LA, McMeekin TA, Ross T (2008) Effect of relative inoculum concentration on *Listeria monocytogenes* growth in co-culture. Int J Food Microbiol 121: 157–168. 18083261 10.1016/j.ijfoodmicro.2007.10.010

[43] Loughman JA, Caparon MG (2007) Comparative functional analysis of the lac operons in *Streptococcus pyogenes* . Mol Microbiol 64: 269–280. 17371500 10.1111/j.1365-2958.2007.05663.x

[44] Trainor VC, Udy RK, Bremer PJ, Cook GM (1999) Survival of *Streptococcus pyogenes* under stress and starvation. FEMS Microbiol Lett 176: 421–428. 10427725 10.1111/j.1574-6968.1999.tb13692.x

[45] Doern GV (1990) *Branhamella catarrhalis*: phenotypic characteristics. Am J Med 88: 33S–35S. 2111091 10.1016/0002-9343(90)90259-g

[46] Upton M, Tagg JR, Wescombe P, Jenkinson HF (2001) Intra- and interspecies signaling between *Streptococcus* salivarius and *Streptococcus pyogenes* mediated by SalA and SalA1 lantibiotic peptides. J Bacteriol 183: 3931–3938. 11395456 10.1128/JB.183.13.3931-3938.2001 PMC95275

[47] Attia AS, Sedillo JL, Hoopman TC, Liu W, Liu L, et al (2009) Identification of a bacteriocin and its cognate immunity factor expressed by *Moraxella catarrhalis* . BMC Microbiol 9: 207. 19781080 10.1186/1471-2180-9-207 PMC2761928

[48] Schaar V, Nordstrom T, Morgelin M, Riesbeck K (2011) *Moraxella catarrhalis* outer membrane vesicles carry beta-lactamase and promote survival of *Streptococcus* pneumoniae and *Haemophilus influenzae* by inactivating amoxicillin. Antimicrob Agents Chemother 55: 3845–3853. 21576428 10.1128/AAC.01772-10 PMC3147650

[49] Armbruster CE, Swords WE (2010) Interspecies bacterial communication as a target for therapy in otitis media. Expert Rev Anti Infect Ther 8: 1067–1070. 20954869 10.1586/eri.10.109 PMC3109636

